# Olfactory performance and odor liking are negatively associated with food neophobia in children aged between 3 and 9 years

**DOI:** 10.1186/s12937-024-01011-6

**Published:** 2024-09-11

**Authors:** Agnieszka Sorokowska, Dominika Chabin, Aleksandra Kamieńska, Sabina Barszcz, Katarzyna Byczyńska, Klaudia Fuławka, Arkadiusz Urbanek, Anna Oleszkiewicz

**Affiliations:** 1 Institute of Psychology, University of Wroclaw, ul. Dawida 1, Wroclaw, 50-527 Poland; 2 Institute of Pedagogy, University of Wroclaw, ul. Dawida 1, Wroclaw, 50-527 Poland; 3 Smell and Taste Clinic, Technische Universität Dresden, Fetscherstr. 74, 01307 Dresden, Germany

**Keywords:** Food neophobia, Child nutrition, Feeding problems, Sensory sensitivity, Olfaction

## Abstract

**Background:**

Child food neophobia, i.e., rejection or avoidance of novel foods at a young age, is a prevalent nutrition problem that affects the quality of children’s diet and impedes the development of healthy food preferences. Sensory sensitivity can relate to the degree of food neophobia, but previous studies rarely focused on the olfactory component of this problem in children.

**Objective:**

We aimed to thoroughly examine the relationship between various aspects of olfactory sensitivity and food neophobia in children.

**Methods:**

246 children aged between three and nine years took part in a food neophobia assessment as well as in a comprehensive, psychophysical olfactory testing.

**Results:**

We found that certain smell perception aspects such as lower odor liking, poorer odor identification ability as well as lower sensitivity to an unknown non-food odor all significantly predicted higher food neophobia in children. Among individual characteristics of either a child or a caregiver, only the child’s age significantly and positively predicted food neophobia. The exploratory model looking into the role of family environment factors predicting self-reported food neophobia in children revealed that food neophobia was associated with lower control given to a child in this child’s feeding process, as well as with a more frequent use of food as a reward in feeding.

**Conclusions:**

We suggest that suppressed olfactory perception and performance can play a unique role in child nutritional difficulties. The study inspires further considerations of olfaction-engaging interventions to counteract food-neophobia in children.

**Supplementary Information:**

The online version contains supplementary material available at 10.1186/s12937-024-01011-6.

## Introduction

Child food neophobia (CFN), i.e., rejection or avoidance of novel foods at a young age [1, 2], is a prevalent problem that affects the quality of children’s diet and impedes the development of healthy food preferences. High food neophobia is one of the strongest psychological barriers to increasing dietary variety and introducing a nutrient-balanced diet in children [3–8]. This feeding problem is categorized within the group of Avoidant/Restrictive Food Intake (ARFID) disorders, which belong to a broader group of sensory food aversions [1, 9, 10].

Unsurprisingly, among a wide range of factors associated with child food neophobia (see e.g., [10, 11], for reviews), existing research consistently points to a significant relationship between the degree of food neophobia and child’s sensory sensitivity. For example, child food neophobia is negatively associated with enjoyment of tactile play [12, 13], and neophobic children often reveal atypical, high sensitivity-related sensory profiles [5, 14]. Olfaction, also known as the sense of smell, which is an important element of human feeding behaviors [15] was also found to relate to food neophobia. However, research on food neophobia and human sensitivity to odors, their perception and assessment has been rather limited, and has mostly involved adult samples. In comparison to adult food neophilics, food neophobics exhibited a lower enjoyment of odors [16–18], and expressed less positive attitudes to olfactory qualities of foods [19]. Further, they had poorer odor identification skills both in an odor identification test [20], and in a task involving identification of spices [18]. Food neophobia in adults showed some links also with olfactory detection abilities. People with food-neophobic tendencies assessed odors as less intense than people without such propensities [17] (but see: [21]), and a study involving individuals with autistic traits showed poorer olfactory detection threshold for a food-related odor (chocolate) in adults exhibiting food neophobia-related behaviors [22]. Finally, high food neophobia predicted a lower overall score in a standardized olfactory test, especially in a discrimination task [23].

There are just a few studies on food neophobia and olfaction in children. They point to a significant association of these two variables, but are not fully consistent with results of studies involving adults in terms of the direction of this relationship. In a parental-report questionnaire study employing parents of 73 children aged 2–5 years [14], high taste/smell sensitivity (analyzed jointly and assessed through questions focused on children’s reactions to various olfactory, gustatory and oral stimuli) predicted higher levels of child food neophobia. Another work, involving a sensory exploration of a novel fruit, showed that food neophobia negatively correlated with a “smells good to eat” rating of this fruit in sixty-eight 5–10 year-olds [24]. These questionnaire and behavioral findings suggesting a relatively high olfactory sensitivity and low appreciation of odors were further supported by the only three existing psychophysical reports. A study among 123 toddlers aged between 20 and 22 months by Monnery-Patris et al. [25] showed that smell reactivity, defined as the relative strength of behavioral responses to 8 odorants, was modestly and positively related to food neophobia in boys. Further, food neophobic children sniffed further away from an odorous food source (an apple or an apple labelled “apple cake”) in a project involving 36 children aged 4–7 [26], and in a study involving non-typically developing children (10 children with autism spectrum disorders aged between 6 and 13 years), high food neophobia was observed in children who disliked odors commonly rated as “nice” [27].

In summary, olfactory experiences are a crucial component of human feeding behaviors and it seems that sensitivity to odors as well as their liking might relate to food neophobia. At the same time, previous studies on food neophobia and the sense of smell (a) are rather scarce, (b) include mostly adults, (c) rarely use psychophysical tests, (d) are not fully consistent in terms of a direction of the relationship between food neophobia and odor perception. In the current study, we aimed to thoroughly and comprehensively analyze the association of food neophobia and smell sensitivity by testing a broad range of olfactory predictors, comprising both questionnaire and psychophysical methods. Further, we measured several additional variables [related to children’s health status, family environment (family feeding practices, SES, caregiver’s education), demographic situation (child’s gender, age, SES) and psychological characteristics (anxiety)], to show a wide and detailed background of the association between olfactory perception and child food neophobia.

## Materials and methods

### Ethics

The study was accepted by the Ethics Committee, Institute of Psychology, University of Wroclaw (20201/EWCKO) and an informed consent to be included in the project was obtained from all participants (orally) as well as their from their caregivers (in writing).

### Participants

#### Sample size estimation

Within the multiple regression design including food neophobia measures, all olfactory sensitivity measures and other variables of interest (see Methods), to obtain power of 0.95 with alpha level et to 0.05 to observe a small effect of *f =* 0.10 according to Cohen’s criteria (1988), the projected sample size was at least 236 subjects. To account for potential data collection issues, the initial sample was to comprise about 260 children.

### Study recruitment

The information on the project was distributed in various education units in Lower Silesia district (Poland). The preliminary study inclusion criteria was child’s age between 3 and 8 years, good overall health condition (to be further verified in a screening questionnaire described in the *Exclusions* section), and participation in the project with at least one primary caregiver (mother/father/legal guardian). The initial sample recruited to the project comprised 273 children, and we obtained an initial caregiver’s written and child’s oral consent to participate in the study from this group.

### Exclusions

Provided the nature of the project, there were certain exclusion criteria related to health or psychological issues of possible importance in the context of the study outcomes. Preliminary questionnaires completed by primary caregivers included a detailed interview on the child: current infections, medication, previous head traumas, chronic diseases (particularly: gastroenterological or smell-related), psychological or psychiatric diagnoses, allergies, and any type of medical problems associated with the sense of smell or food consumption. Severe health disorders, chronic diseases, developmental disabilities, autism spectrum disorder or predetermined food intake disorders, as well as a complete loss of smell were treated as exclusion criteria.

The preliminary screening questionnaires completed by the caregivers revealed that five children suffered from severe health conditions: cancer (*n =* 1), Asperger’s syndrome (*n =* 1), Non-typical autism spectrum disorder (*n =* 1), cerebral palsy (*n =* 1), Down’s syndrome (*n =* 1). Their data were excluded from our final analyses. It was also impossible to perform the food neophobia testing with 14 children (e.g., some of them were unable to focus for a prolonged time, some refused to continue study participation, some were repeatedly absent from preschool/school). Additionally, despite repeated contact attempts, 8 parents did not complete the final survey on their children. As food neophobia was the main variable of interest, inability to obtain the food neophobia score from either the child or the parent resulted in participant’s data not being included in the final analyses.

Some among the children retained in the final sample were reported to suffer from current infections (self-report: *n =* 31), chronic diseases (*n =* 43; mostly allergies *n* = 28), to have some dietary restraints (*n =* 31) or to take medication (*n =* 19). However, as indicated by *t*-tests, their olfactory perception indices as well as their food neophobia scores did not differ significantly from the scores of the children with no such problems/health issues (all *p*s > 0.05). Therefore, their data were retained in the final analyses. Two children recruited and tested in school groups in the sample proved to be slightly above the planned age threshold (they were already 9 at the time of the study) – but since they met all other project inclusion criteria we decided to use their data in the final analyses.

#### Final sample

For the sample inclusion and exclusion flowchart, see **Supplementary Figure ****S1**. The final study sample comprised 246 children aged 3–9 years (boys: *n* = 119, *M*_*age*_=5.49 ± 0.1.2; girls: *n* = 127, *M*_*age*_=5.43 ± 0.10). Age distribution was similar across genders, *t*(244) = 0.36, *p* = .72 [95% CI: − 0.25; 0.35].

## Methods

### Food neophobia

The child food neophobia was measured through children’s self-assessments and caregiver-completed assessments. For children’s self-report we used an abbreviated, 8-item Food Neophobia Scale [28]. Following Laureati et al. [28], we applied a 5-item pictorial scale for the self-assessments. Caregiver’s report about a child was performed with the same, 8-item Food Neophobia Scale [28], but using a full, original, Likert-type, 1 to 7 response scale. The primary analyses were performed using the child’s self-assessment, but all analyses were also repeated using the caregiver assessment of the child’s food neophobia level (see *Statistical analyses* section).

### Olfactory perception

The analyzed olfactory perception indices included odor significance and awareness, odor identification score, hedonic evaluation of odors (odor liking), as well as two types of thresholds for odor detection that were assessed using the methods described in the following paragraphs.

*Odor significance and awareness.* Awareness of surrounding odors and their importance in children’s daily life was assessed by means of an odor significance and awareness questionnaire based on COBEL scale by Ferdenzi and collaborators [29]. As we needed a brief, simple and quantitative instrument, we selected 9 items of the original scale that targeted three different areas of functioning – social, environmental and food-related. The items were slightly modified to apply a forced-response format. We used 3 items in social component [items number 12, 13, 14, e.g., *Do you find that people smell of something*,* even without perfume or deodorant (no/yes some people/yes everyone)*?], 3 items in environment component [items number 2, 3, 4, e.g., *Do you remember odors you smelled yesterday (food odors not acceptable)? Which ones?*], 3 items in food component [items number 1, 2, 16, e.g., *When you smell a food odor*,* do you try to guess for fun what it is (never/sometimes/often)*?]; item number 2 (original version: *Imagine your parents present you a dish you do not know: will you do something before putting it in your mouth (yes/no)? What do you do? Will you smell it (yes/no)?)* was divided into two questions, for the sake of simplicity and to better capture feeding-related smelling behaviors: *I smell food before I try it (Often/sometimes/never)* and *When I am presented with a dish I do not know*,* I smell it before trying it (Often/sometimes/never)*]. Therefore, our final *odor significance and awareness* measure had 10 items with forced-choice responses which were further coded as 0-1-2, yielding an average score that ranged between 0 and 2 (0 indicating lower odor significance and awareness and 2 – higher).

*Odor identification and odor liking assessments.* Odor identification ability is a capacity to recognize and correctly name different odors [30]. In the current study, it was assessed with the U-Sniff test [31] – a method designed specifically for children and adapted in the participants’ country of origin. The participants were asked to choose a name of an odor presented by an experimenter from a list of four of alternatives. The final identification score was a sum of correct responses (range 0 to 12). Prior to odor identification, the children were asked to assess the pleasantness of each smell using the aforementioned 1 to 5 pictorial scale that had been applied in the food neophobia measurement [28]. The pleasantness perception score (henceforth named *odor liking*) was computed as a mean of all pleasantness assessments made by a child.

*Detection thresholds.* Olfactory threshold is the lowest concentration at which an odor is reliably detected [32]. One of the most popular tools used to measure this olfactory ability is the threshold subtest of the Sniffin’ Sticks Test (SST; [30]). Here, we used two threshold tasks, both based on an odor highly unfamiliar to children. The first was a “classic”, commercially available Sniffin’ Sticks test containing various n-butanol concentrations [30]. The second, custom-made test, contained 16 solutions of a highly unfamiliar food odor (ginger; natural ginger oil by Sigma-Aldrich/Merck group) diluted in commercially available propylene glycol (Sigma-Aldrich/Merck Group). A standard, single-staircase, three alternative forced choice (3-AFC) procedure in ascending order of concentration was used (from 16-lowest concentration, lowest threshold to 1-highest concentration, highest threshold). In each concentration step a participant was presented with three dispensers, one of which contained the target odor – his/her task was to point to this target dispenser [30]. The final threshold for odor detection score ranges between 1 and 16, with a higher score indicating higher sensitivity (or – in other words – a lower threshold for odor detection) [see [30] for a detailed procedure of threshold score assessment and calculation].

### Additional predictors

There are certain demographic factors and individual differences that may relate to child food neophobia. Caregiver’s higher food neophobia [33], lower socio-economic status [34, 35], or lower education [36, 37] tend to positively predict child’s food neophobia. Further, men and women may differ in the degree of food neophobia, although research is rather inconsistent with regard to the direction of this difference [FN higher in men: [35, 38]; FN higher in women: [17], no gender differences [5, 37, 39, 40]. Furthermore, food neophobia seems to be negatively related with temperamental approach tendencies [41], and positively with anxiety [42].

To control for the potential impact of these predictors on our results, we additionally collected data on several variables of interest in the caregivers’ survey: child’s age, gender and anxiety, family socio-economic status (SES), caregiver’s age, education (number of completed years of education) and caregiver’s food neophobia. We additionally included child’s BMI to the controlled variables to account for potential nutritional problems often associated with child food neophobia. Caregivers assessed the family SES in comparison to the average in one’s country using a Likert-type scale ranging from 0 (much worse than the average) to 10 (much better than the average). Child’s anxiety was tested using the Generalized Anxiety Disorder subscale (6 items) derived from the Spence Children’s Anxiety Scale – Parent version (SCAS-Parent), with a response scale ranging from 0 (never) to 4 (always) [43]. Primary caregiver’s food neophobia was tested with the full, 10-item Food Neophobia Scale [2].

### Exploratory analyses

Provided the recent findings in the area of food neophobia research, we also decided to examine family feeding practices to perform an exploratory analysis of the relative contribution of family environment to the child food neophobia. Family Feeding Practices were assessed using the Comprehensive Feeding Practices Questionnaire (CFPQ; [44]). The scale consists of 12 factors that can be further clustered in three groups targeting controlling feeding practices (pressure to eat, restriction for health, and restriction for weight), feeding practices promoting autonomy (healthy environment, encourage balance and variety, teaching nutrition, monitoring, modeling, involvement and child control) and practices focused on applying food for non-nutritive purposes (emotion regulation and food as a reward). Respondents provide their answers using Likert-type, 1 to 5 scales, with answers ranging from “never” to “always” for “child control”, “emotion regulation”, and “monitoring” factors and responses ranging from “disagree” to “agree” for nine remaining factors. Higher scores represent using more of a corresponding feeding practice, or using it more frequently.

### Procedure

The information on the project was distributed in various preschools and schools, and prior to the testing phase, the team members visited each school and preschool included in the project to conduct olfactory workshops, familiarizing themselves with the children and establishing personal contact with teachers. After an initial caregiver’s written and child’s oral consent to participate in the study was obtained, a given family was invited to the further parts of the project. The caregiver questionnaires and study invitations were distributed through school and preschool teachers.

The testing was performed by trained research team members. Two individual testing sessions took place in designated rooms in preschools and schools of the participants. The first testing session typically involved the food neophobia assessment, one threshold for odor detection test, as well as odor identification and odor liking tasks. During the second testing session, the children completed the odor awareness scale and the remaining threshold test. Each testing session took between 20 and 30 min. Whenever it was desired by a child, a familiar teacher accompanied it during the testing sessions.

### Statistical analyses

Data were analyzed with IBM SPSS software (version 29; Armonk, NY: IBM Corp) with a significance level set to α = 0.05. Child food neophobia scores were distributed normally according to the Shapiro-Wilk’s coefficient, both for child’s self-report (*p* = .11) and parent/caregiver report (*p* = .06). We assessed the consistency of reports of food neophobia obtained from parent and child with Pearson’s *r* correlations and paired-samples t-test. In these initial comparisons, food neophobia report scores were converted to a percentage scale due to the different range of Likert scales in the child (5-point) and parent (7-point) questionnaires. For the more intuitive presentation of the results, further analyses were conducted with the average scores (not percentages). We assessed the reliability of the responses in the child food neophobia questionnaires provided by the parents/caregivers and children with Cronbach’s α.

To compare all scores of interest between children high and low in food neophobia, we divided children into high-neophobic and low-neophobic based on quartiles in child food neophobia scores. Children scoring in the first quartile were categorized as low-food neophobic and children whose score was in the fourth quartile were assigned to high-food neophobic group [see [23]]. This division was again made for self- and parent/caregiver-reported food neophobia. Between these two groups (high and low in food neophobia), we compared odor awareness, odor identification, odor liking, and thresholds for butanol and ginger odors, while controlling for age with general linear model (GLM). Following the peer-review process, to streamline the results section, these results are presented in the supplementary materials (**Sect. 2**).

In the main analysis, performed for the entire study sample, to assess the predictive value of a set of psychological and olfactory factors in estimating the level of child food neophobia, we constructed two hierarchical regression models (separately for self- and parent/caregiver-reported food neophobia). Hierarchical regression models contained the following blocks: [1] the child’s individual characteristics (age, sex, BMI) [2], the parent’s/caregiver’s characteristics (food-neophobia, education, socioeconomic status) [3], child’s anxiety [4], olfactory abilities (odor awareness, odor identification, odor liking, thresholds for butanol and ginger odors). Finally, in a supplementary analysis, we regressed the subscales of the Comprehensive Feeding Practices Questionnaire [CFPQ; [44]] on self- and parent/caregiver-reported food neophobia in two separate regression models.

### Discrepancies between pre-registration and analysis of observed data

The study has been preregistered at https://aspredicted.org/LXN_BHL. Overall, we have followed analyses path declared in the preregistration. To improve clarity and for the sake of brevity we have replaced LMMs with hierarchical regression models. This solution served setting the exploration of the relationship between olfactory performance and perception and food neophobia in the context of previously examined variables, such as child’s and parent’s/caregiver’ characteristics and psychological underpinnings (anxiety). As a consequence, we resigned from presenting correlations between olfactory sensitivity and food neophobia to avoid repetitive analyses and inflation of the inference risk of error due to multiple testing. Given the high correlation between child’s and parent/caregiver’s age we removed the latter variable from the tested models. The remaining pre-registered analyses have been implemented and reported. As indicated above, following the peer-review process, the GLM analysis was moved to the supplementary materials.

## Results

### Descriptive statistics

All data for this study are available at https://osf.io/xmfn6/?view_only=d0d744660e8a421caf65ea2a804309bd and detailed descriptive statistics for the main variables included in the study can be found in Supplementary Table S1.

On average, parents assessed their children as more food neophobic (*M* = 58.9 ± 1) than children assessed themselves (*M* = 52.7 ± 0.85), *t*(245) = 5.43, *p* < .001 [95% CI: 3.92, 8.4]. The reliability of the self-reported food neophobia in children was α = 0.50, and that of food neophobia reports obtained from the parents was α = 0.88. The distributions of child-assessed food neophobia and parental report on child food neophobia are depicted in Supplementary Figure S2 and Supplementary Figure S3 provides a visualization of the convergence in food neophobia scores based on assessments performed by children and their caregivers. Following the approach proposed in the pre-registration, we also compared the olfactory perception scores between children classified as high in food neophobia and low in food neophobia. Supplementary Table S2 provides an illustration of the convergence in classification of children as low, average or high in food neophobia based on children’s self-assessments and caregiver assessments. The results of the analyses based on these classifications are presented in the Supplementary Materials (**Sect. 2**; Supplementary Figure S4, Table S3 and Table S4).

Food neophobia reported by the children and their parents/caregivers was moderately and positively correlated (*r* = .26, *p* < .001; see also scatterplot in Figure S3). Based on this relatively low convergence between children’s and caregivers’ reports, we decided to focus on predicting child-reported food neophobia in the subsequent analyses and move regression models predicting parent/caregiver-reported neophobia to the Supplementary Materials (see Figure S3 and Table S2). Using children’s self-reports appeared more plausible given the changes in eating behaviors and the increasing feeding independence of children with age, as well as provided the potential decline in parents’ knowledge about their children’s feeding-related behaviors.

### Predicting self-reported food neophobia in children

Each block of the hierarchical regression model proved to significantly predict self-reported food-neophobia in children (all *F*s > 2.23, all *p* < .04). In the children’s individual characteristics block, age was the only significant predictor of food neophobia (β = 0.24, *p* = .001) showing an increase in food neophobia with age. Neither the parent’s/caregiver’s characteristics nor the child’s anxiety blocks yielded any additional significant predictors, but adding olfactory performance as a final block in our model significantly improved data fitness (*p* = < 0.001) and showed that lower olfactory performance was associated with greater self-reported food neophobia. Specifically, we found lower odor identification (β=-0.23, *p* = .004), lower odor liking (β=-0.18, *p* = .01), and poorer odor detection threshold for n-butanol (β=-0.19, *p* = .01) to predict self-reported food neophobia. Altogether the model explained 13.1% of variance as indicated by the adjusted R^2^. All model coefficients can be found in the Supplementary Table S5. The analysis based on caregiver-reported food neophobia can also be found in the Supplement (for detailed results see Supplementary Table S6).

The exploratory model looking into the role of family environment factors predicting self-reported food neophobia in children revealed that food neophobia was associated with lower control given to a child in this child’s feeding process (β=-0.15, *p* = .03), as well as with a more frequent use of food as a reward in feeding (β = 0.16, *p* = .047). Altogether, family environment factors explained 5% of variance in self-reported food neophobia in children. All hierarchical regression coefficients for this analysis are presented in the Supplementary Table S7. The analysis based on caregiver-reported food neophobia can be found in the Supplement (for detailed results see Supplementary Table S8).

## Discussion

Food neophobia is a prevalent and difficult problem affecting lives of parents and children worldwide [45–48]. Creating a barrier to increasing a child’s dietary variety [3], food neophobia can potentially drive poorer health outcomes in children. Regardless of any evolutionary benefits it may have once been associated with, food neophobia currently constitutes an important nutritional problem. Therefore, it seems worthy to understand its causes and correlates, and potentially also to address them in further interventions. In the current study, we aimed to explore the olfactory-related sensory components of food neophobia. We found that lower odor liking, poorer olfactory identification ability as well as lower sensitivity to an unknown non-food odor all significantly predicted higher food neophobia in children. Additionally, we observed that some feeding practices, such as using food as a reward and exerting higher parental control over the feeding process were more frequent in families of children higher in food neophobia. We therefore provide insights into the role of olfactory perception and performance, as well as family feeding habits in shaping food neophobia.

The most important finding of the current project is that higher self-reported food neophobia was associated with lowered odor liking, poorer olfactory identification, and decreased detection ability for butanol. Our main regression analysis had an advantage of employing a large sample of children varying in food neophobia level, which concurs with a recommendation not to overuse extreme group analysis in food neophobia research [49]. Our findings are in line with the previous observations that higher food neophobia predicts a less positive assessment of a fruit odor [24] as well as with distancing behaviors from food-related odors [26]. However, they rather contrast the reports on a positive association of child food neophobia with taste/smell sensitivity assessed through questionnaires [14] and higher responsiveness to odors in food-neophobic toddlers observed in a behavioral task [25]. Further, research focused on sensory modalities other than olfaction also tended to document a rather enhanced sensory sensitivity in food-neophobic children [1]. Food neophobia was moreover found to predict a certain over-sensitivity to what can be defined as “early warning” cues in feeding behavior [19, 50], such as visual aspects of unfamiliar foods [51]. Olfaction is a “distal” sense alongside vision, and – like vision – it also enables to assess stimuli without touching them. Our results not only show no olfactory over-sensitivity, but even suggest a lower smell sensitivity in food neophobic children. We may propose at least two plausible explanations of this seeming, interesting incongruence.

First, olfaction may need to be trained to achieve expertise. Such a training in its formalized form is based on a structured, daily exposure to a predetermined set of odors [52]. However, even everyday olfactory experiences, active search for olfactory cues, reliance on odors and their appreciation all likely enrich the smell expertise [53]. This type of olfactory learning in food-neophobic children may be hindered by low odor pleasantness perception and a consequent, increased avoidance/weariness of various odor sources. In this understanding, lower liking of odors we observed could be a determinant of neophobic behaviors, similar to a dislike of other sensory characteristics of foods reported in former studies [1]. This hypothetical explanation aligns also with a report indicating that food neophobia negatively correlated with a “smells good to eat” rating in 5–10 year-olds [24]. Expanding the olfactory perceptiveness of children characterized with food-neophobic tendencies can be additionally hindered by two other important aspects of their functioning – cognitive rigidness and their tendency to react with high arousal to various stimuli. For example, a series of studies involving various cross-categorization tasks indicated a cognitive rigidity in utilizing knowledge structures by food neophobic children [54, 55] and their difficulties with changing perspectives once items have been classified [56]. Further, high food neophobia was shown to be predictive of an increased arousal response in reaction to various qualities of food, such as higher taste intensity, flavor complexity, its perceived dangerousness, or “foreignness” [57]. It is likely that such an intense reaction would be also generated by various qualities of odors in children with high levels of food neophobia. Such a strong (and likely negative) arousal in response to unfamiliar odors (or maybe even to familiar, but disliked ones), combined with an inability to modify this reaction may contribute to a slower olfactory development.

Second, we measured olfactory expertise using standardized tools for olfactory diagnosis. As such, these tests require an active participation and keen engagement of the subjects in the smell tasks, which includes also a deep inhalation of the presented odors. At the same time, disgust- or anxiety-evoking experiences can lead to a considerably shallower inhalation, or even to a short cessation of breathing in a presence of a negative stimulus [58, 59]. Smell test scores in people who do not enjoy odors in general may consequently be lower. Olfactory behaviors were indeed found to be cautious in food-neophobics – they were reported to exhibit lower sniff magnitude, less exploratory behaviors [16], and sniffing further away from foods or food-named odor samples [26]. Such avoidant behaviors, mirroring the breathing pattern characteristic of a disgust response, might have actually decreased the performance in both the identification and in the threshold tasks in our study. Further, persistence of the avoidant breathing pattern in daily “smell environment” might again lead to a less intense olfactory development, consistent with the reasoning presented above and previous literature on odor exposure effects [60, 61]. These hypotheses cannot be tested using our data, since we did not control for sniff intensity (deepness of inhalation) of our participants. However, a longitudinal design including breathing pattern and sniff intensity assessment in children of different ages, varying in liking of odors and in food neophobia levels, would be ideal to test these assumptions and to observe the direction of the potential effects. It would also be recommendable to investigate whether other sensory correlates of food neophobia contribute to this problem in a similar way, exploring the effect sizes and directions of the associations.

The outcomes of our study inspire further considerations of olfaction-engaging interventions to counteract food-neophobia in children. Food consumption encompasses not only the act of tasting but also the multisensory exploration of food (for a review see [62]). Research has demonstrated that complex, sensory-targeted experimental interventions can significantly assist children in overcoming food neophobia [63–66]. Olfaction contributes to food perception, acceptance and enjoyment [15, 17, 67], making it a viable channel for interventions aimed at modifying feeding-related behaviors. Importantly, olfactory abilities, including those in children [68]), can be enhanced through targeted olfactory training [52]. Moreover, such training has been shown to affect children’s functioning in non-olfactory domains, like emotional categorization [69]. Thus, it would be highly valuable to investigate the potential effectiveness of targeted olfactory training on food neophobia. There are several possible ways a structured olfactory exposure could benefit children. Contact with a variety of odors in a controlled environment might reduce their overall arousal response to odors or increase general odor liking, ultimately leading to fewer food-rejecting behaviors. Focusing on the olfactory component alone could additionally enhance our understanding of how different sensory modalities contribute to the effectiveness of interventions aimed at reducing food neophobia. However, it is crucial to note that the proposed mechanisms are purely hypothetical, and the relationships observed in our study do not imply causality. It is also possible that food neophobia results in lower olfactory expertise rather than the reverse. If this is the case, the effectiveness of olfactory training on food neophobia may be limited.

In the current study, we performed an exploratory analysis of the role of family feeding practices in food neophobia, and we observed more frequent rewards and lower child’s control in feeding of children of higher food neophobia. It has previously been shown that using rewards in attempt to encourage the children to eat may be counter effective [70–72], mitigating the positive food exposure effects – our results underscore and reconfirm this conclusion. At the same time, feeding practices focused on empowering children may positively affect their nutrition [73], and a positive example from a caregiver may encourage a child to food-neophilic behaviors [42, 74]. Although this was not directly related to the sensory angle of our study, this exploratory analysis showed that family environment factors explained 5% of variance in self-reported food neophobia in children. Obviously, it should be remembered that the observed associations may again be driven both by child food neophobia influencing feeding practices, as well as by parental behaviors promoting the development of food-neophobic behaviors. Nevertheless, it seems worthy to draw on our broad analysis to guide future holistic intervention strategies sensitizing caregivers and educators to various factors potentially affecting food-neophobic behaviors in children.

In our research, age was the only significant individual characteristic significantly predicting self-assessed food neophobia, and we observed an increase in food neophobia with age in our sample of children. Previous studies generally indicate that the level of this feeding problem is the highest in children between 2 and 6 years of age [1, 75, 76]. After this sensitive period, food neophobia often decreases to reach a relative stability later in life [with some studies pointing to adolescence [77] and some – to adulthood [78]]. Our study indicates that the level of food neophobia can remain on a similar level, or even increase, in older children. It is intriguing to investigate why, despite increasing experience and safe exposure to foods as children age, the level of food neophobia in our sample did not decrease. One possibility is that elementary school pupils, as they gain relative independence in their eating habits, may still prefer to avoid unfamiliar foods, thus exhibiting higher food neophobia. Another, more hypothetical explanation could be that younger children are less neophobic than older children, potentially indicating a slight “generational difference” in feeding approaches. Teachers and parents have become increasingly aware of the importance of healthy eating and often promote a positive attitude towards healthy foods in children through practices such as baby-led weaning (BLW) or involving young children in meal planning and preparation. The very recent rise in popularity of these approaches may lead to gradual changes in food attitudes between older and younger children. Overall, at the current stage of research it is hard to explain our result definitively. A recommended approach would involve further investigations comparing food neophobia scores among significantly older and younger children relative to the age ranges observed in our study, as well as conducting longitudinal studies. Additionally, it would be valuable to supplement this research with behavioral assessments of food neophobia (see e.g., [64]), to further empirically validate the findings. We also encourage future readers of our paper to conduct additional analyses and explore various hypotheses using our database. For instance, non-linear assessments of the association between food neophobia and age, or using pre-determined age categories in the analyses could enhance the understanding of our outcomes.

### Limitations and future directions

Whilst the findings of this research add to the important body of knowledge concerning the predictors of food neophobia, our study has some limitations. There are several questionnaires used to measure food neophobia [see [79] for a review]. The scale developed by Pliner and Hobden [2] seems to be one of the most commonly used in the literature [80] and can be applied in research involving children following certain modifications [28]. However, in our study, the reliability of the self-reported food neophobia in children was rather low (α = 0.50). Such response consistency is in line with the literature on questionnaire self-assessments in children [81] and does not need to indicate low data quality. Still, the children’s self-assessments and parental reports were not entirely coherent in our sample. While we did observe a positive correlation between the two variables, it was rather low. One reason for this discrepancy might be a different understanding of the item contents. In caregiver assessments, food neophobia was strongly related to anxiety, indicating a potentially different angle of assessments, or perhaps even an overlap or a certain bias in perception of the caregivers. It seems that an optimal solution for future studies would be supplementing the assessments with behavioral food neophobia testing (see e.g., [64]).

There were also other inconsistencies in the results observed in our study. For instance, we aimed to measure children’s ability to detect both a food-related odor (ginger) and a non-food-related odor (n-butanol) in relation to food neophobia. We found that only the n-butanol detection threshold significantly predicted food neophobia, and this was true solely for self-assessed food neophobia. While this result might suggest potential issues with the odor detection task in our sample, we propose an alternative interpretation. The experimenters had no doubts regarding the intelligibility of the threshold test to the participants. Also, as illustrated in the Supplementary Figure S5, distributions of threshold scores across age groups raise no particular concerns for proficiency of even young children in this test. It could thus be considered why the two measured odor detection thresholds could be differently related to food neophobia in our sample. One reason may be that ginger was a much more pleasant or more familiar odor than butanol, masking the effects we expected to observe in our study. However, it also cannot be excluded that the custom-made ginger detection test we prepared had some flaws, like a potential trigeminal component helping its detection by food-neophobic children (of otherwise poorer olfactory abilities). We performed no actual verification of validity of this measure prior to our experiment. Overall, we would again suggest supplementing the further experiments with other methods, allowing for a broader assessment of sensory sensitivity (e.g., using neurophysiological assessments).

Finally, it should be noted that some results we observed in relation to children’s self-assessments were not observed when parental reports were analyzed. This was found both for the olfactory indices and family feeding practices. The most consistent effects, observed across measures and different types of analyses, was a significant association of food neophobia with odor identification abilities and child control factor in the feeding practices group. We would like to highlight the potential importance of these two predictors, and to underscore that regardless of some potential issues, our data still indicate that both sensory and family environment components significantly relate to food neophobia.

## Conclusion

We found that olfactory sensitivity, odor identification and odor liking were significantly and negatively related to food neophobia. The exploratory model looking into the role of family environment factors predicting self-reported food neophobia in children additionally revealed that food neophobia was associated with lower control given to a child in this child’s feeding process, as well as with a more frequent use of food as a reward in feeding. These findings underscore the importance of considering both sensory and environmental factors when addressing food neophobia in children, providing a foundation for developing targeted interventions to mitigate this prevalent issue.

## Electronic supplementary material

Below is the link to the electronic supplementary material.

Supplementary Material 1

## Data Availability

All data for this study are available at https://osf.io/xmfn6/?view_only=d0d744660e8a421caf65ea2a804309bd.

## Declarations

### Preregistration

The study has been preregistered at https://aspredicted.org/LXN_BHL.
