# Correction: Olfactory performance and odor liking are negatively associated with food neophobia in children aged between 3 and 9 years

**DOI:** 10.1186/s12937-024-01015-2

**Published:** 2024-09-27

**Authors:** Agnieszka Sorokowska, Dominika Chabin, Aleksandra Kamieńska, Sabina Barszcz, Katarzyna Byczyńska, Klaudia Fuławka, Arkadiusz Urbanek, Anna Oleszkiewicz

**Affiliations:** 1 Institute of Psychology, University of Wroclaw, ul. Dawida 1, Wroclaw, 50-527 Poland; 2 Institute of Pedagogy, University of Wroclaw, ul. Dawida 1, Wroclaw, 50-527 Poland; 3 Smell and Taste Clinic, Technische Universität Dresden, Fetscherstr. 74, 01307 Dresden, Germany

**Correction: Nutr J 23 105 (2024)**

10.1186/s12937-024-01011-6

Following publication of the original article [1], the author reported that given names and family names of all authors were interchanged.

This has been corrected above and the original article has been updated.
