# Supplementary material for: Olfactory performance and odor liking are negatively associated with food neophobia in children aged between 3 and 9 years
Source: Nutr J. 2024 Sep 11;23:105. doi: 10.1186/s12937-024-01011-6 (PMC11389506; doi:10.1186/s12937-024-01011-6)
Supplement: Supplementary file 1 — Supplementary Material 1 [file 12937_2024_1011_MOESM1_ESM.docx]

**Supplementary Materials**

**Section 1. Descriptive statistics**

**Figure S1**

Sample recruitment flowchart

- - N=273 children

Recruitment

- - Cancer (*n=*1)
  - Asperger’s syndrome (*n=*1)
  - Non-typical autism spectrum disorder (*n=*1)
  - Cerebral palsy (*n=*1)
  - Down’s syndrome (*n=*1)

Exclusions

- - Missing parent data (n=8)
  - CFN testing impossible (n=14)

Missing data

- - N=246 children

Final sample

**Table S1**

Descriptive statistics for main variables included in the study (child and caregiver reports)

|  | N | Mean | SE | SD | Min | Max |
| --- | --- | --- | --- | --- | --- | --- |
| Odor significance and awareness [0-2] | 232 | 0.90 | 0.026 | 0.395 | 0.00 | 1.89 |
| Odor identification [1-12] | 244 | 7.17 | 0.15 | 2.34 | 1.00 | 12.00 |
| Odor liking [1-5] | 244 | 3.53 | 0.042 | 0.652 | 1.17 | 5.00 |
| Odor threshold (n-butanol) [1-16] | 237 | 8.80 | 0.292 | 4.497 | 0.50 | 16.00 |
| Odor threshold (ginger) [1-16] | 223 | 8.96 | 0.260 | 3.883 | 1.00 | 16.00 |
| Child-reported food neophobia [1-5] | 246 | 2.63 | 0.043 | 0.670 | 1.00 | 4.38 |
| Child-reported general neophobia [1-5] | 185 | 3.16 | 0.057 | 0.779 | 1.00 | 5.00 |
| *Caregiver reports* |  |  |  |  |  |  |
| Caregiver-reported child food neophobia [1-7] | 246 | 4.12 | 0.070 | 1.100 | 1.13 | 7.00 |
| Caregiver-reported child general neophobia [1-7] | 186 | 3.28 | 0.071 | 0.966 | 1.00 | 6.33 |
| Child anxiety [0-4] | 245 | 1.40 | 0.046 | 0.718 | 0.00 | 3.00 |
| Body mass index (child) | 241 | 15.12 | 0.128 | 1.963 | 9.72 | 24.79 |
| *Caregiver self-reports* |  |  |  |  |  |  |
| Caregiver-reported food neophobia [1-7] | 245 | 3.26 | 0.066 | 1.028 | 1.00 | 6.50 |
| Caregiver-reported general neophobia [1-7] | 184 | 3.08 | 0.083 | 1.123 | 1.00 | 6.33 |
| Caregiver age [years] | 243 | 36.91 | 0.290 | 4.520 | 26.00 | 48.00 |
| Caregiver education [years] | 235 | 16.56 | 0.197 | 3.026 | 6 | 30 |
| Family socio-economic status [0-10] | 212 | 6.42 | 0.102 | 1.485 | 2 | 10 |

**Figure S2**

Distributions of food neophobia scores in children’s self-assessments (panel A) and in parental assessments (panel B).

*
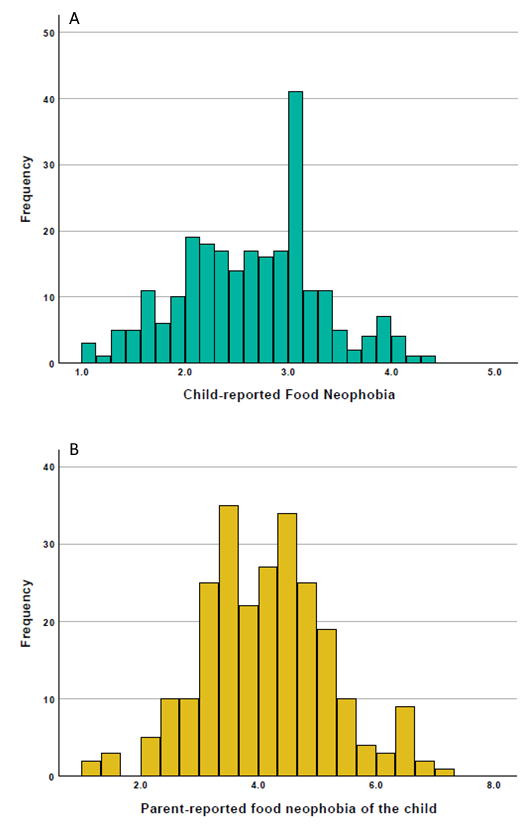
*

**Figure S3**

Scatterplot illustrating the convergence in food neophobia scores based on assessments performed by children and their caregivers.

**Section 2. Olfactory performance in children with low/high food neophobia**

Children were qualified as low in self-reported food neophobia if their score was =<2.25 points (1^st^ quartile; n=78) and high in self-reported food neophobia with the score >=3.125 (4^th^ quartile; n=63). Based on the parent/caregiver reports on the child’s food neophobia we qualified children as low in neophobia if their score was =<3.375 points (1^st^ quartile; n=67) and high in food neophobia if their score was >=4.78 points (4^th^ quartile; n=61). The classification based on children’s self-reports and parent/caregiver-reported data was not fully consistent. The table below (Table S2) illustrates the overlap (number of classifications) between food neophobia categories based on assessments performed by children and caregivers.

**Table S2**

Number of children classified as low, average and high in food neophobia based on assessments performed by children and their caregivers.

|  |  | Caregivers’ assessment of child food neophobia | | |  |
| --- | --- | --- | --- | --- | --- |
|  |  | Low | Average | High | Total count |
| Self-assessed child food neophobia | Low | 31 | 29 | 18 | 78 |
|  | Average | 28 | 52 | 25 | 105 |
|  | High | 8 | 37 | 18 | 63 |
| Total count |  | 67 | 118 | 61 | 246 |

***Section 2a.*** *Olfactory performance in children with low/high self-assessed food neophobia*

Children assessing their food neophobia as low identified more odors (*M*=.62±.02) than children self-assessing their food neophobia as high (*M*=.52±.02), *F*(1,126)=9.81; *p*=.002. There were no other significant differences in olfactory performance between children high or low in self-reported food neophobia (see **Figure S4** and **Table S3**).

**Figure S4**

Chemosensory performance and perception across children declared to be high vs low food neophobic.

Age was a significant covariate for odor awareness, odor identification abilities and for threshold for butanol detection (all *p*s<.05). All GLM coefficients for the analysis based on children’s self-reports are presented below (**Table S3**).

**Table S3**

GLM coefficients comparing chemosensory perception and performance in children who described themselves as high vs low food-neophobic.

| Source | Dependent Variable | Type III Sum of Squares | df | Mean Square | *F* | Sig. |
| --- | --- | --- | --- | --- | --- | --- |
| Corrected Model | Odor awareness | .363^a^ | 2 | .181 | 1.358 | .261 |
|  | Odor identification | 1.055^b^ | 2 | .527 | 16.987 | <.001 |
|  | Odor liking | 4.281^c^ | 2 | 2.141 | 4.393 | .014 |
|  | Odor threshold (n-butanol) | 172.638^d^ | 2 | 86.319 | 4.679 | .011 |
|  | Odor threshold (ginger) | 9.059^e^ | 2 | 4.530 | .296 | .744 |
| Intercept | Odor awareness | 3.334 | 1 | 3.334 | 24.960 | <.001 |
|  | Odor identification | .155 | 1 | .155 | 4.998 | .027 |
|  | Odor liking | 94.713 | 1 | 94.713 | 194.362 | <.001 |
|  | Odor threshold (n-butanol) | 44.348 | 1 | 44.348 | 2.404 | .124 |
|  | Odor threshold (ginger) | 341.159 | 1 | 341.159 | 22.322 | <.001 |
| Age | Odor awareness | .002 | 1 | .002 | .016 | .901 |
|  | Odor identification | .945 | 1 | .945 | 30.439 | <.001 |
|  | Odor liking | 2.278 | 1 | 2.278 | 4.676 | .033 |
|  | Odor threshold (n-butanol) | 169.210 | 1 | 169.210 | 9.173 | .003 |
|  | Odor threshold (ginger) | 7.754 | 1 | 7.754 | .507 | .478 |
| Child-reported food neophobia | Odor awareness | .353 | 1 | .353 | 2.644 | .107 |
|  | Odor identification | .305 | 1 | .305 | 9.810 | .002 |
|  | Odor liking | 1.036 | 1 | 1.036 | 2.126 | .147 |
|  | Odor threshold (n-butanol) | 23.786 | 1 | 23.786 | 1.289 | .258 |
|  | Odor threshold (ginger) | .204 | 1 | .204 | .013 | .908 |
| Error | Odor awareness | 16.430 | 123 | .134 |  |  |
|  | Odor identification | 3.818 | 123 | .031 |  |  |
|  | Odor liking | 59.938 | 123 | .487 |  |  |
|  | Odor threshold (n-butanol) | 2269.038 | 123 | 18.447 |  |  |
|  | Odor threshold (ginger) | 1879.856 | 123 | 15.283 |  |  |
| Total | Odor awareness | 97.806 | 126 |  |  |  |
|  | Odor identification | 46.669 | 126 |  |  |  |
|  | Odor liking | 1637.742 | 126 |  |  |  |
|  | Odor threshold (n-butanol) | 11100.250 | 126 |  |  |  |
|  | Odor threshold (ginger) | 12212.250 | 126 |  |  |  |
| Corrected Total | Odor awareness | 16.792 | 125 |  |  |  |
|  | Odor identification | 4.873 | 125 |  |  |  |
|  | Odor liking | 64.220 | 125 |  |  |  |
|  | Odor threshold (n-butanol) | 2441.677 | 125 |  |  |  |
|  | Odor threshold (ginger) | 1888.915 | 125 |  |  |  |
| a. R Squared = .022 (Adjusted R Squared = .006) | | | | | | |
| b. R Squared = .216 (Adjusted R Squared = .204) | | | | | | |
| c. R Squared = .067 (Adjusted R Squared = .051) | | | | | | |
| d. R Squared = .071 (Adjusted R Squared = .056) | | | | | | |
| e. R Squared = .005 (Adjusted R Squared = -.011) | | | | | | |

***Section 2b.*** *Olfactory performance in children with low/high parent/caregiver-reported food neophobia – results and model coefficients*

Mirroring the findings on children’s self-reported food neophobia, we found a significant advantage of children described as low in food neophobia (*M*=.64±.02) in odor identification over children assessed as highly neophobic (*M*=.55±.02), *F*(1,107)=6.85; *p*=.01. For all model coefficients see **Table S4**.

**Table S4**

GLM coefficients comparing chemosensory perception and performance in children who have been described as high vs low food neophobic by their parent/caregiver

| Source | Dependent Variable | Type III Sum of Squares | df | Mean Square | *F* | Sig. |
| --- | --- | --- | --- | --- | --- | --- |
| Corrected Model | Odor awareness | .220^a^ | 2 | .110 | 1.006 | .369 |
|  | Odor identification | .799^b^ | 2 | .400 | 13.475 | <.001 |
|  | Odor liking | .161^c^ | 2 | .081 | .220 | .803 |
|  | Odor threshold (n-butanol) | 166.122^d^ | 2 | 83.061 | 3.952 | .022 |
|  | Odor threshold (ginger) | 5.507^e^ | 2 | 2.753 | .187 | .829 |
| Intercept | Odor awareness | 2.352 | 1 | 2.352 | 21.482 | <.001 |
|  | Odor identification | .167 | 1 | .167 | 5.631 | .019 |
|  | Odor liking | 60.454 | 1 | 60.454 | 165.068 | <.001 |
|  | Odor threshold (n-butanol) | 25.351 | 1 | 25.351 | 1.206 | .275 |
|  | Odor threshold (ginger) | 262.517 | 1 | 262.517 | 17.871 | <.001 |
| Age | Odor awareness | .035 | 1 | .035 | .316 | .575 |
|  | Odor identification | .722 | 1 | .722 | 24.340 | <.001 |
|  | Odor liking | .143 | 1 | .143 | .390 | .534 |
|  | Odor threshold (n-butanol) | 166.021 | 1 | 166.021 | 7.900 | .006 |
|  | Odor threshold (ginger) | 5.456 | 1 | 5.456 | .371 | .544 |
| Parent/caregiver-reported food neophobia | Odor awareness | .146 | 1 | .146 | 1.335 | .251 |
|  | Odor identification | .203 | 1 | .203 | 6.849 | .010 |
|  | Odor liking | .003 | 1 | .003 | .008 | .930 |
|  | Odor threshold (n-butanol) | 9.122 | 1 | 9.122 | .434 | .511 |
|  | Odor threshold (ginger) | .074 | 1 | .074 | .005 | .944 |
| Error | Odor awareness | 11.715 | 107 | .109 |  |  |
|  | Odor identification | 3.174 | 107 | .030 |  |  |
|  | Odor liking | 39.188 | 107 | .366 |  |  |
|  | Odor threshold (n-butanol) | 2248.696 | 107 | 21.016 |  |  |
|  | Odor threshold (ginger) | 1571.757 | 107 | 14.689 |  |  |
| Total | Odor awareness | 86.740 | 110 |  |  |  |
|  | Odor identification | 43.677 | 110 |  |  |  |
|  | Odor liking | 1443.431 | 110 |  |  |  |
|  | Odor threshold (n-butanol) | 10404.875 | 110 |  |  |  |
|  | Odor threshold (ginger) | 10326.000 | 110 |  |  |  |
| Corrected Total | Odor awareness | 11.935 | 109 |  |  |  |
|  | Odor identification | 3.973 | 109 |  |  |  |
|  | Odor liking | 39.349 | 109 |  |  |  |
|  | Odor threshold (n-butanol) | 2414.818 | 109 |  |  |  |
|  | Odor threshold (ginger) | 1577.264 | 109 |  |  |  |
| a. R Squared = .018 (Adjusted R Squared = .000) | | | | | | |
| b. R Squared = .201 (Adjusted R Squared = .186) | | | | | | |
| c. R Squared = .004 (Adjusted R Squared = -.015) | | | | | | |
| d. R Squared = .069 (Adjusted R Squared = .051) | | | | | | |
| e. R Squared = .003 (Adjusted R Squared = -.015) | | | | | | |

**Section 3. Predictors of food neophobia in children: regression analyses**

***Section 3a.*** *Predicting self-reported food neophobia in children – regression coefficients*

**Table S5**

Regression coefficients describing the predictive value of child’s and parent/caregiver’s characteristics, anxiety and chemosensory perception and performance for child-reported food neophobia

| Model | | Unstandardized Coefficients | | Standardized Coefficients | *t* | Sig. |
| --- | --- | --- | --- | --- | --- | --- |
|  |  | B | Std. Error | Beta |  |  |
| 1 | (Constant) | 2.107 | .431 |  | 4.885 | <.001 |
|  | Child's age | .138 | .042 | .237 | 3.307 | .001 |
|  | Child's s gender | .006 | .095 | .005 | .066 | .948 |
|  | Child's BMI | -.014 | .024 | -.040 | -.561 | .575 |
| 2 | (Constant) | 2.132 | .552 |  | 3.862 | <.001 |
|  | Child's age | .131 | .042 | .225 | 3.132 | .002 |
|  | Child's gender | .010 | .095 | .007 | .101 | .919 |
|  | Child's BMI | -.014 | .024 | -.041 | -.576 | .565 |
|  | Parent/caregiver food neophobia | .083 | .045 | .134 | 1.837 | .068 |
|  | Parent/caregiver's education | -.012 | .016 | -.055 | -.752 | .453 |
|  | Family socioeconomic status | -.007 | .032 | -.016 | -.225 | .822 |
| 3 | (Constant) | 2.147 | .555 |  | 3.869 | <.001 |
|  | Child's age | .134 | .043 | .230 | 3.139 | .002 |
|  | Child's gender | .009 | .095 | .007 | .093 | .926 |
|  | Child's BMI | -.015 | .024 | -.043 | -.599 | .550 |
|  | Parent/caregiver food neophobia | .085 | .046 | .136 | 1.858 | .065 |
|  | Parent/caregiver's education | -.012 | .016 | -.052 | -.714 | .476 |
|  | Family socioeconomic status | -.007 | .032 | -.015 | -.210 | .834 |
|  | Anxiety | -.025 | .069 | -.027 | -.366 | .715 |
| 4 | (Constant) | 3.094 | .613 |  | 5.050 | <.001 |
|  | Child's age | .200 | .046 | .343 | 4.340 | <.001 |
|  | Child's s gender | .041 | .092 | .031 | .443 | .658 |
|  | Child's BMI | -.015 | .023 | -.044 | -.635 | .526 |
|  | Parent/caregiver food neophobia | .075 | .045 | .120 | 1.681 | .095 |
|  | Parent/caregiver's education | -.012 | .016 | -.053 | -.750 | .454 |
|  | Family socioeconomic status | -.010 | .031 | -.022 | -.317 | .752 |
|  | Anxiety | -.016 | .067 | -.017 | -.238 | .812 |
|  | Odor awareness | -.054 | .135 | -.028 | -.395 | .693 |
|  | Odor identification | -.789 | .268 | -.226 | -2.940 | .004 |
|  | Odor liking | -.186 | .074 | -.182 | -2.506 | .013 |
|  | Odor threshold (n-butanol) | -.027 | .010 | -.186 | -2.562 | .011 |
|  | Odor threshold (ginger) | .012 | .012 | .071 | 1.009 | .314 |
| a. Dependent Variable: child-reported food-neophobia | | | | | | |

***Section 3b.*** *Predicting parent/caregivers-reported food neophobia in children*

Although none of the blocks in the hierarchical regression model significantly fit the observed parent/caregiver-reported food neophobia data (all *F*s<1.62, all *p*s>.14), certain predictors were significantly related to food neophobia. Among individual characteristics of the child, we noted an increase in food neophobia with age (β=.15, *p*=.043). None of the parent characteristics were relevant for the assessment of the child’s food neophobia. Adding anxiety in the third block did not improve our predictions, however, anxiety turned out a significant predictor in the fourth block (β=.15, *p*=.046) and so did odor identification (β=-.19, *p*=.02).

**Table S6**

Regression coefficients describing the predictive value of child’s and parent/caregiver’s characteristics, anxiety and chemosensory perception and performance for parent/caregiver-reported food neophobia

| Model | | Unstandardized Coefficients | | Standardized Coefficients | *t* | Sig. |
| --- | --- | --- | --- | --- | --- | --- |
|  |  | B | Std. Error | Beta |  |  |
| 1 | (Constant) | 3.814 | .743 |  | 5.133 | <.001 |
|  | Child's age | .147 | .072 | .148 | 2.037 | .043 |
|  | Child's gender | .065 | .163 | .029 | .401 | .689 |
|  | Child's BMI | -.037 | .042 | -.065 | -.887 | .376 |
| 2 | (Constant) | 3.460 | .961 |  | 3.601 | <.001 |
|  | Child's age | .141 | .073 | .143 | 1.939 | .054 |
|  | Child's gender | .078 | .165 | .035 | .475 | .635 |
|  | Child's BMI | -.033 | .042 | -.057 | -.777 | .438 |
|  | Parent/caregiver's food neophobia | .019 | .079 | .018 | .240 | .811 |
|  | Parent/caregiver's education | .026 | .028 | .068 | .913 | .363 |
|  | Family socioeconomic status | -.027 | .056 | -.037 | -.491 | .624 |
| 3 | (Constant) | 3.340 | .958 |  | 3.487 | <.001 |
|  | Child's age | .118 | .074 | .120 | 1.602 | .111 |
|  | Child's gender | .085 | .164 | .038 | .516 | .607 |
|  | Child's BMI | -.028 | .042 | -.048 | -.657 | .512 |
|  | Parent/caregiver's food neophobia | .007 | .079 | .007 | .088 | .930 |
|  | Parent/caregiver's education | .021 | .028 | .056 | .756 | .451 |
|  | Family socioeconomic status | -.031 | .056 | -.042 | -.562 | .575 |
|  | Child's anxiety | .209 | .119 | .131 | 1.755 | .081 |
| 4 | (Constant) | 3.335 | 1.099 |  | 3.035 | .003 |
|  | Child's age | .206 | .083 | .208 | 2.489 | .014 |
|  | Child's gender | .080 | .165 | .036 | .484 | .629 |
|  | Child's BMI | -.029 | .042 | -.052 | -.703 | .483 |
|  | Parent/caregiver's food neophobia | -.022 | .080 | -.021 | -.273 | .785 |
|  | Parent/caregiver's education | .025 | .028 | .066 | .881 | .380 |
|  | Family socioeconomic status | -.029 | .056 | -.038 | -.514 | .608 |
|  | Child's anxiety | .242 | .120 | .152 | 2.011 | .046 |
|  | Odor awareness | .249 | .243 | .077 | 1.026 | .306 |
|  | Odor identification | -1.109 | .481 | -.188 | -2.307 | .022 |
|  | Odor liking | .002 | .133 | .001 | .012 | .990 |
|  | Odor threshold (n-butanol) | -.021 | .019 | -.086 | -1.125 | .262 |
|  | Odor threshold (ginger) | .016 | .021 | .055 | .743 | .458 |
| a. Dependent Variable: Parent/caregiver-reported food neophobia | | | | | | |

**Section 4. Family feeding practices and food neophobia in children: regression analyses**

***Section 4a.*** *Family feeding practices and self-reported food neophobia in children – regression coefficients*

**Table S7**

*Family feeding practices and child food neophobia (children’s self-assessments).*

| Model | | Unstandardized Coefficients | | Standardized Coefficients | *t* | Sig. |
| --- | --- | --- | --- | --- | --- | --- |
|  |  | B | Std. Error | Beta |  |  |
| 1 | (Constant) | 3.157 | .404 |  | 7.809 | <.001 |
|  | Child control | -.181 | .082 | -.150 | -2.220 | .027 |
|  | Emotion regulation | -.033 | .062 | -.035 | -.525 | .600 |
|  | Encourage balance and variety | -.114 | .080 | -.119 | -1.427 | .155 |
|  | Healthy environment | .096 | .080 | .102 | 1.196 | .233 |
|  | Food as a reward | .117 | .058 | .156 | 1.996 | .047 |
|  | Involvement | -.122 | .072 | -.125 | -1.682 | .094 |
|  | Modeling | -.112 | .068 | -.125 | -1.635 | .103 |
|  | Monitoring | .035 | .044 | .054 | .797 | .427 |
|  | Pressure to eat | .105 | .068 | .132 | 1.547 | .123 |
|  | Restriction for health | .032 | .066 | .039 | .485 | .628 |
|  | Restriction for weight | -.081 | .074 | -.082 | -1.096 | .274 |
|  | Teaching nutrition | .065 | .079 | .059 | .822 | .412 |
| a. Dependent Variable: child-reported food-neophobia | | | | | | |

***Section 4b.*** *Family feeding practices and parent/caregivers-reported food neophobia in children – results and regression coefficients*

Among family environment factors we found child control (β=.18, *p*=.006) to be linked with greater food neophobia reported by the parents/caregivers. Family environment factors were responsible for explaining 8% of variance in parent/caregiver reported food neophobia in children.

**Table S8**

*Family feeding practices and child food neophobia (caregiver assessments).*

| Model | | Unstandardized Coefficients | | Standardized Coefficients | *t* | Sig. |
| --- | --- | --- | --- | --- | --- | --- |
|  |  | B | Std. Error | Beta |  |  |
| 1 | (Constant) | 3.526 | .656 |  | 5.371 | <.001 |
|  | Child control | .367 | .133 | .183 | 2.767 | .006 |
|  | Emotion regulation | .046 | .101 | .030 | .457 | .648 |
|  | Encourage balance and variety | -.125 | .129 | -.079 | -.964 | .336 |
|  | Healthy environment | -.129 | .130 | -.083 | -.993 | .322 |
|  | Food as a reward | .057 | .095 | .047 | .605 | .546 |
|  | Involvement | -.215 | .117 | -.133 | -1.830 | .069 |
|  | Modeling | -.062 | .111 | -.042 | -.560 | .576 |
|  | Monitoring | -.016 | .072 | -.015 | -.218 | .827 |
|  | Pressure to eat | .194 | .110 | .148 | 1.765 | .079 |
|  | Restriction for health | .184 | .107 | .137 | 1.721 | .087 |
|  | Restriction for weight | -.102 | .120 | -.062 | -.851 | .395 |
|  | Teaching nutrition | .227 | .129 | .125 | 1.760 | .080 |
| a. Dependent Variable: Parent/caregiver-reported food neophobia | | | | | | |

**Section 5. Additional data visualisations**

**Figure S5**

Distributions of detection thresholds scores for n-butanol (panel A) and ginger odor (panel B) in children varying in age.

**
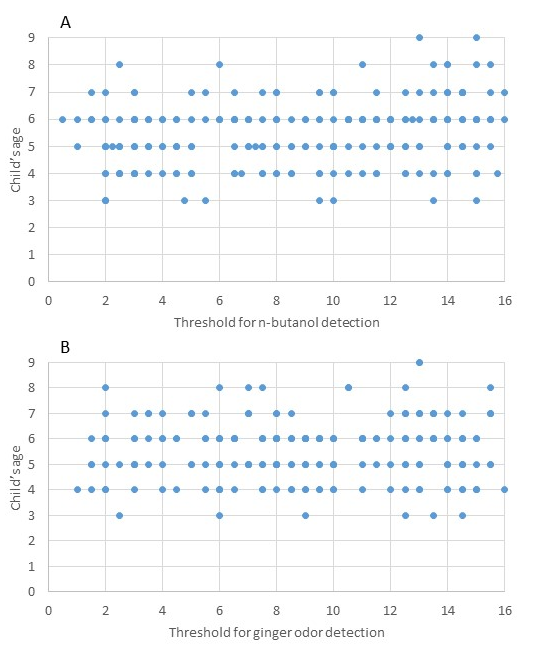
**
